# Supplementary figures and images for: Production of cytotoxic compounds in dedifferentiated cells of Jatropha curcas L. (Euphorbiaceae)
Source: PeerJ. 2016 Nov 1;4:e2616. doi: 10.7717/peerj.2616 (PMC5101598; doi:10.7717/peerj.2616)

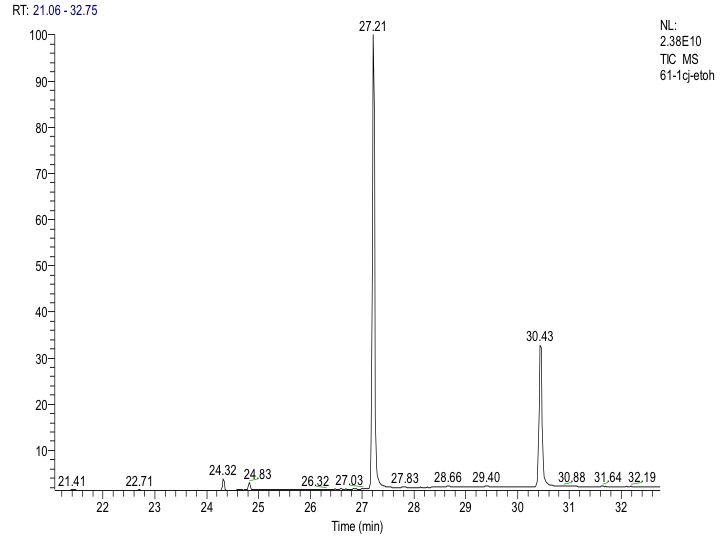

Supplement: Figure S1 — A major component (stilbene-like) is shown with retention time of 27.21 min [file peerj-04-2616-s001.jpg]
